# Supplementary material for: MicroRNAs in Plasma-Derived Extracellular Vesicles as Non-Invasive Biomarkers for Eosinophilic Esophagitis
Source: Int J Mol Sci. 2025 Jan 14;26(2):639. doi: 10.3390/ijms26020639 (PMC11766357; doi:10.3390/ijms26020639)
Supplement: Supplementary file 1 [file ijms-26-00639-s001.zip › ijms-3415421-supplementary -updated.pdf]

International Journal of
Molecular Sciences

MDPI

Supplementary Material

MicroRNAs in Plasma-Derived Extracellular Vesicles as Non-Invasive Biomarkers for Eosinophilic Esophagitis

Elena Grueso-Navarro [1,2,3,*], Leticia Rodríguez-Alcolado [1,2,3,4], Laura Arias-González [1,2,3,5], Ana M. Aransay [2,6], Juan-José Lozano [2], Julia Sidorova [2], Rocío Juárez-Tosina [7], Jesús González-Cervera [3,8], Alfredo J. Lucendo [1,2,3,5], and Emilio J. Laserna-Mendieta [1,2,3,5]

1 Department of Gastroenterology, Hospital General de Tomelloso, 13700 Tomelloso, Spain; ajlucendo@hotmail.com (A.J.L.); ejlaserna@sescam.jccm.es (E.J.L.-M.)
2 Centro de Investigación Biomédica en Red de Enfermedades Hepáticas y Digestivas (CIBERehd), Instituto de Salud Carlos III, 28029 Madrid, Spain
3 Instituto de Investigación Sanitaria de Castilla-La Mancha (IDISCAM), 45071 Toledo, Spain
4 Department of Surgery, Medical and Social Sciences, Universidad de Alcalá, 28805 Alcalá de Henares, Spain
5 Instituto de Investigación Sanitaria Princesa, 28006 Madrid, Spain
6 Center for Cooperate Research in Biosciences (CIC bioGUNE), Basque Research and Technology Alliance (BRTA), 48160 Derio, Spain
7 Department of Pathology, Hospital General La Mancha Centro, 13600 Alcázar de San Juan, Spain
8 Department of Allergy, Hospital General de Tomelloso, 13700 Tomelloso, Spain
* Correspondence: egrueson@sescam.jccm.es

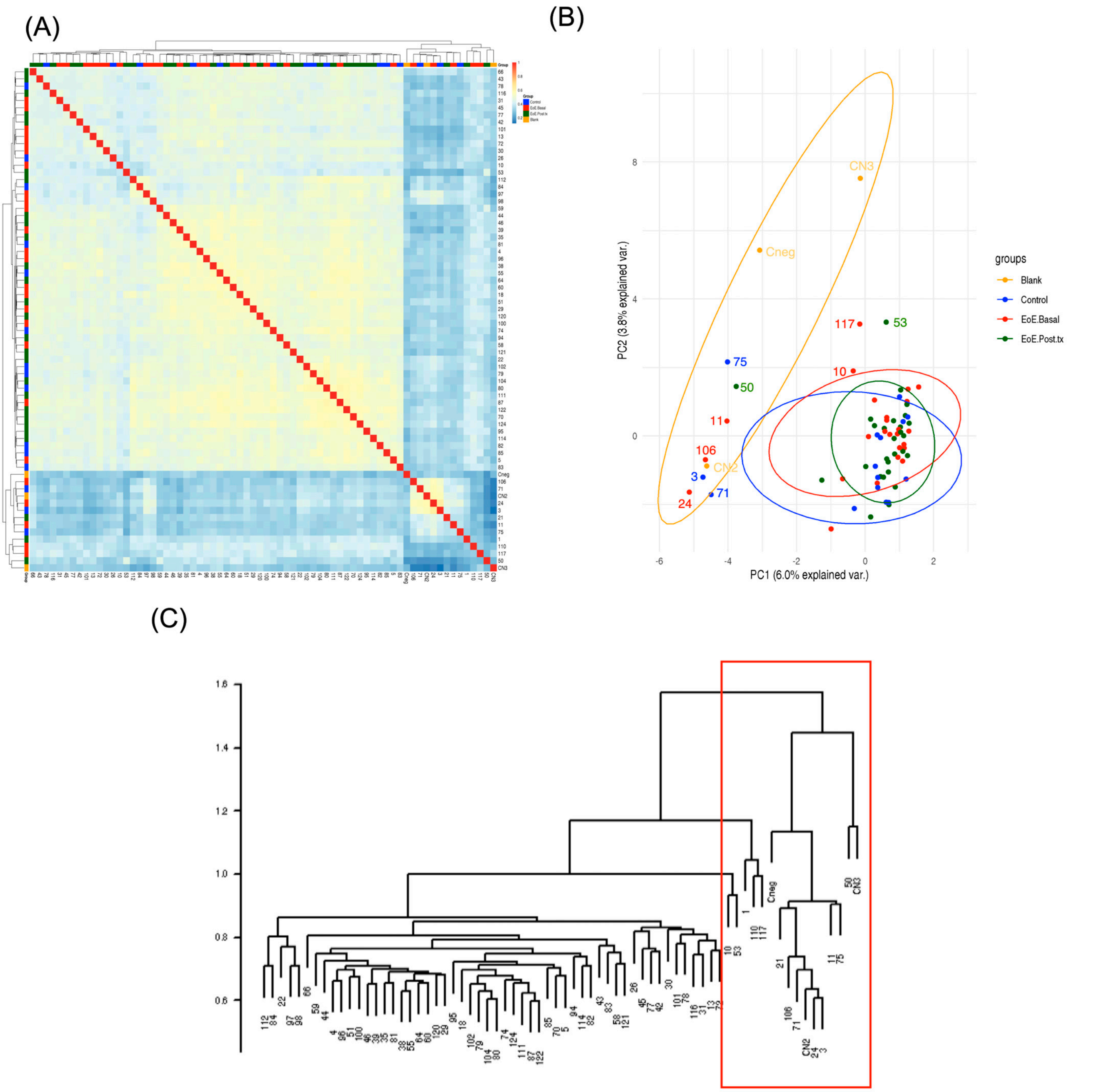

Figure S1. Quality control analysis of small-RNA-seq data. A) Heatmap plot showing the correlation of the three negative controls used with 11 of the samples from the discovery cohort, according to their expression profile. B) Principal component analysis (PCoA) plot showing correlation analysis. C) Dendrogram from unsupervised correlation analysis associated 13 samples with negative controls.

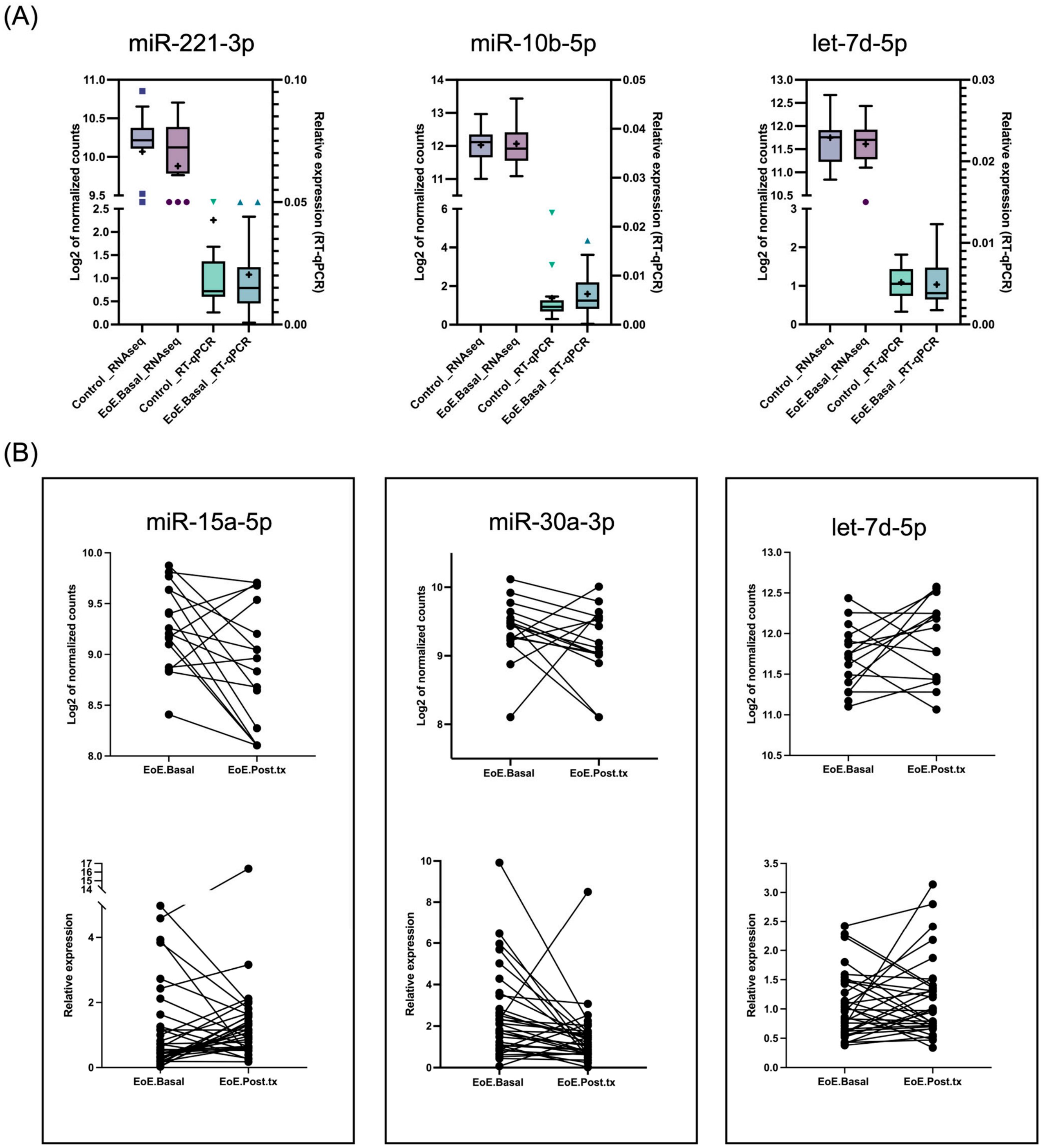

Figure S2. Comparing sRNA expression trends between conditions measured by RNAseq and RT-qPCR. A) Box and whisker plot (Tukey's method) showing the median (horizontal bar) and IQR of miR-221-3p, miR-10b-5p and let-7d-5p expression in EVs from Controls vs. EoE.Basal. Horizontal line shows the median. Mean value is indicated by "+". Values outside IQR are displayed as individual points. EoE.Basal-RNAseq, n=19, EoE.Basal-RTqPCR, n=33; controls-RNAseq, n=11; controls-RTqPCR, n=14. B) Before-and-after plots showing expression of miR-15a-5p, miR-30a-3p and let-7d-5p measured by RNAseq and RT-qPCR in EVs from EoE patients before (EoE.Basal) and after treatment (EoE.Post.tx). EoE pairs, n=16.

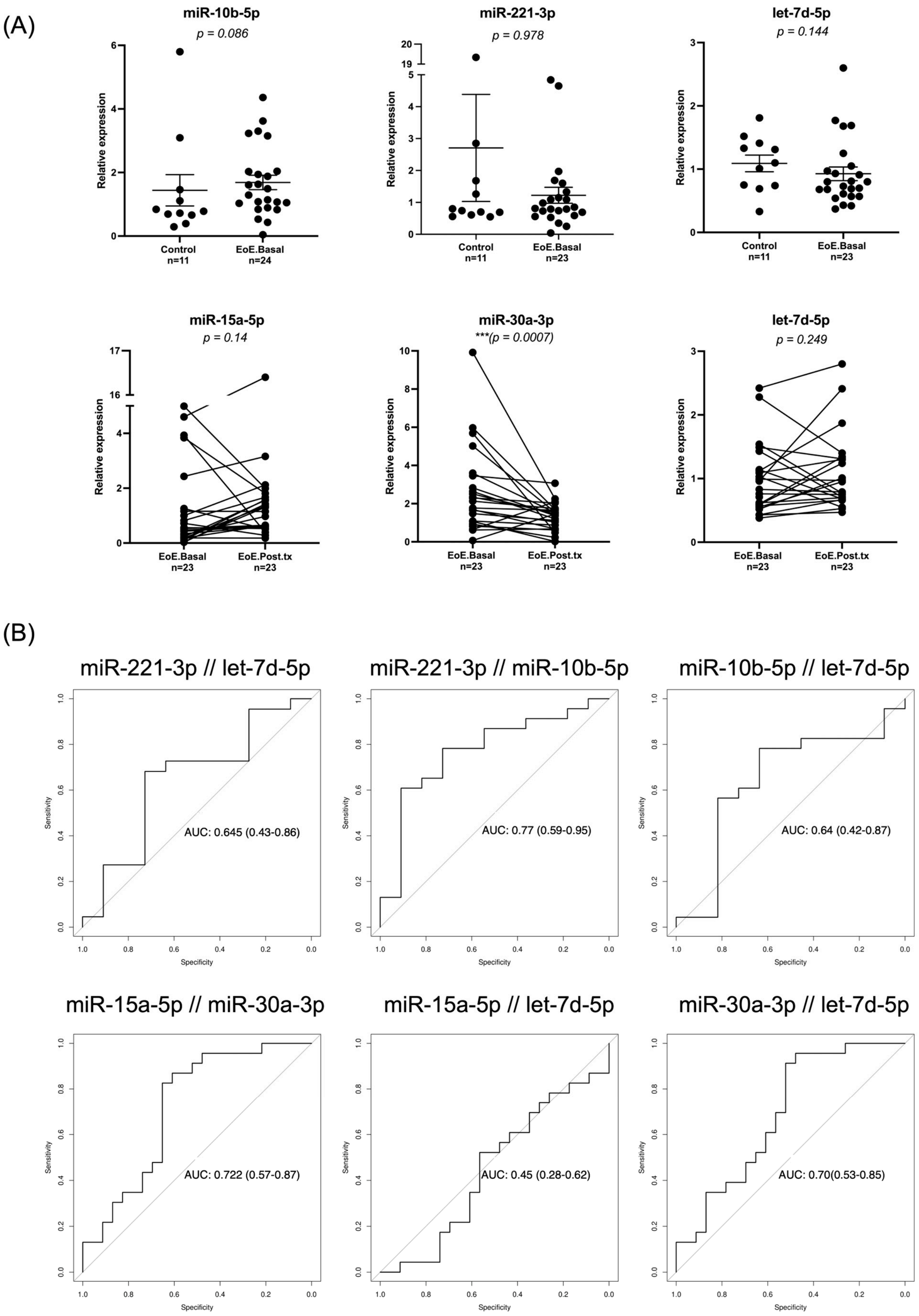

Figure S3. Restricting validation of selected candidates to those patients aged over 18. A) validation by RT-qPCR of selected miRNAs (miR-10b-5p, miR-221-3p, let7-d-5p, miR-15a-5p and miR30a-3p) in adults (aged over 18 years) of the validation cohort. Normalized to let-7a-5p. B) Area under the ROC curve (AUC) analysis of pairwise combinations in adults only.

International Journal of *Molecular Sciences*

MDPI

Table S1: Samples included in the discovery cohort. 4-FED: four-food elimination diet; Bud: budesonide; DE: distal oesophagus; EoE: eosinophilic esophagitis; eos: eosinophils; FP: fluticasone propionate; GERD: gastroesophageal reflux disease; ODT: oral disintegrating tablets; Ome: omeprazole; OVSMPC: oral viscous solution marketed by a pharma company; OVSP: oral viscous solution prepared by a pharmacist; PE: proximal oesophagus; post-tx: post-treatment; PPI: proton pump inhibitors; STC: swallowed topical corticosteroids; Rab: rabeprazole.

Condi-tion	Patient code	Sample ID		Gender	Age	Effective treatment (EoE)/Reason for en-doscopy (controls)	Days between basal & post.tx endoscopies	Peak of eos in PE		Peak of eos in DE		EREFS	
		Basal	Post.tx					Basal	Post.tx	Basal	Post.tx	Basal	Post.tx
EoE	EoE1	11[a]	22[b]	Male	35	PPI, Rab 40 mg/day	63	130	1	80	3	4	0
EoE	EoE3	10[a]	21[a]	Female	39	PPI, Ome 40 mg/day	64	15	0	35	12	4	0
EoE	EoE5	24[a]	38[b]	Male	16	STC, Bud OVSMPC	92	17	0	100	0	3	0
EoE	EoE6	25[a]	42[b]	Male	17	STC, Bud OVSMPC	93	80	8	45	1	5	0
EoE	EoE7	5	43	Male	30	4-FED	273	-	0	35	0	2	0
EoE	EoE9	31	50[a]	Male	14	STC, Bud OVSMPC	90	120	0	100	0	4	0
EoE	EoE10	4	35	Male	21	STC, Bud OVSP	247	60	5	60	14	4	0
EoE	EoE13	30	46	Male	37	4-FED	84	50	0	100	5	6	1
EoE	EoE14	13	51	Male	43	PPI, Rab 40 mg/day	224	46	0	54	0	6	0
EoE	EoE15	18	44	Female	16	STC, Bud OVSMPC	156	40	7	30	2	8	0
EoE	EoE16	39	53[a]	Male	14	STC, Bud OVSMPC	92	50	0	32	7	2	0
EoE	EoE18	45	64	Female	14	STC, Bud OVSMPC	195	30	0	40	0	3	0
EoE	EoE19	29	66	Male	41	4-FED	288	60	2	65	2	3	0
EoE	EoE20	59	70	Female	24	PPI, Ome 40 mg/day	108	0	0	28	2	2	0
EoE	EoE21	72	94	Male	42	STC, FP ODT	97	110	0	100	0	4	0
EoE	EoE22	60	77	Male	22	STC, FP ODT	106	50	0	50	0	5	1
EoE	EoE24	87	95	Male	23	PPI, Ome 40 mg/day	84	11	0	28	0	0	0
EoE	EoE25	96	104	Male	35	STC, FP ODT	95	25	0	28	1	4	0
EoE	EoE26	100	111	Male	16	STC, Bud OVSMPC	88	22	0	7	0	2	0
EoE	EoE27	97	112	Male	36	STC, Bud ODT	132	2	0	23	0	4	0
EoE	EoE28	58	114	Male	23	STC, FP ODT	381	50	0	70	0	4	0
EoE	EoE29	106[a]	116[b]	Male	37	STC, Bud ODT	54	103	0	46	0	5	0
EoE	EoE31	98	122	Male	8	STC, Bud OVSMPC	173	15	0	30	0	3	0
EoE	EoE32	110[a]	121[b]	Male	45	STC, Bud ODT	52	86	0	60	0	5	0

EoE	EoE33	101	120	Male	16	STC, Bud OVSMPC	92	54	0	40	2	6	1
EoE	EoE34	117[a]	124[b]	Female	55	STC, Bud ODT	50	54	0	60	0	3	0
Control	C1	1[a]		Male	44	Dysphagia		0		0			
Control	C2	3[a]		Male	41	GERD		0		0			
Control	C3	26		Male	53	GERD		1		0			
Control	C5	55		Male	19	Dysphagia		1		0			
Control	C8	71[a]		Male	23	GERD		0		0			
Control	C9	74		Male	14	Epigastric pain		-		-			
Control	C10	75[a]		Male	36	Dyspepsia		0		0			
Control	C12	78		Male	20	Epigastric pain		0		0			
Control	C13	79		Male	20	Dysphagia		0		0			
Control	C14	80		Female	36	GERD		0		0			
Control	C15	81		Male	29	GERD		0		0			
Control	C16	82		Female	43	Dyspepsia		-		-			
Control	C17	83		Male	39	GERD		-		-			
Control	C18	84		Female	19	Dyspepsia		0		0			
Control	C19	85		Male	40	GERD		0		0			
Control	C21	102		Female	24	Dyspepsia		0		0			

[a]The samples excluded after quality control analysis. [b]The samples excluded because they were paired to basal samples eliminated after QC analysis

International Journal of *Molecular Sciences*

MDPI

Table S2: List and reasons for sample exclusion from the final RNA-Seq bioinformatic analysis. NC: technical negative control; QC: quality control; tx: treatment.

Sample ID	Group	Reason for exclusion
1	Control patient	Heat-map grouping with NC
3	Control patient	Heat-map grouping with NC & QC failed
10	EoE.Basal/Patient #3	Not passed correlation analysis
11	EoE.Basal/Patient #1	Heat-map grouping with NC
21	EoE.Post.tx/Patient #3	Heat-map grouping with NC
24	EoE.Basal/Patient #5	Heat-map grouping with NC & QC failed
25	EoE.Basal/Patient #6	Not library generated
50	EoE.Post.tx/Patient #9	Heat-map grouping with NC
53	EoE.Post.tx/Patient #16	Not passed correlation analysis
71	Control patient	Heat-map grouping with NC
75	Control patient	Heat-map grouping with NC
106	EoE.Basal/Patient #29	Heat-map grouping with NC & QC failed
110	EoE.Basal/Patient #32	Heat-map grouping with NC
117	EoE.Basal/Patient #34	Heat-map grouping with NC

Table S3: Target genes of miR-30a-3p

Gene Symbol	p-value	FDR
STAU1	0,000129	0,0448
TPR	0,000219	0,0448
NBPF11	0,000597	0,0448
FBLN1	0,00077	0,0448
KMT2A	0,00181	0,0656
C5orf51	0,00204	0,0656
SGSH	0,00231	0,0656
MAATS1	0,00384	0,0656

SLC7A6	0,00384	0,0656
GABRA2	0,00461	0,0656
ZNF418	0,00461	0,0656
KRT7	0,00538	0,0656
MEF2A	0,00615	0,0656
PYROXD1	0,00615	0,0656
KCTD6	0,00692	0,0656
TNFSF13B	0,00692	0,0656
TUBA1A	0,00692	0,0656
VWC2L	0,00692	0,0656
ATP6V0E2	0,00768	0,0656
KIAA1324L	0,00768	0,0656
SULT1C4	0,00768	0,0656
XBP1	0,00768	0,0656
C1QBP	0,00845	0,0656
NBPF1	0,00845	0,0656
ZNF587B	0,00845	0,0656
ARL2BP	0,00921	0,0656
COX17	0,00921	0,0656
LRRC42	0,00921	0,0656

AGGF1	0,00998	0,0656
CACNA1E	0,00998	0,0656
NEMP2	0,00998	0,0656
CCDC25	0,0107	0,0656
SCP2	0,0107	0,0656
SELENOT	0,0107	0,0656
MAML1	0,0123	0,0656
PPARD	0,0123	0,0656
RTP4	0,0123	0,0656
KRTAP5-9	0,013	0,0656
NBPF14	0,013	0,0656
NBPF15	0,013	0,0656
C6	0,0138	0,0656
CDK13	0,0138	0,0656
FAM107B	0,0138	0,0656
MORC3	0,0138	0,0656
SOS2	0,0146	0,066
BECN1	0,0153	0,066
PAIP2	0,0153	0,066
NUFIP2	0,0161	0,066

FBXO40	0,0169	0,066
GSTO1	0,0169	0,066
ATF3	0,0184	0,066
DNAJC30	0,0191	0,066
NBPF10	0,0191	0,066
NBPF12	0,0191	0,066
PCSK2	0,0191	0,066
PPP1R2	0,0199	0,066
ADORA2B	0,0207	0,066
AKAP17A	0,0207	0,066
ATP8B3	0,0207	0,066
C12orf73	0,0207	0,066
GNAL	0,0207	0,066
HBEGF	0,0207	0,066
ITGA5	0,0207	0,066
NBPF3	0,0214	0,0666
RUNX2	0,0214	0,0666
TMEM242	0,0222	0,0676
EPGN	0,023	0,0677
RAB31	0,023	0,0677

PDE11A	0,0237	0,068
ETV6	0,0245	0,068
CYR61	0,0252	0,068
RASGRP3	0,0252	0,068
CCDC86	0,026	0,068
KIAA1671	0,026	0,068
LY6K	0,026	0,068
YWHAG	0,026	0,068
CAMSAP1	0,0275	0,068
EPB41L4B	0,0275	0,068
NFATC3	0,0275	0,068
VPS37A	0,0275	0,068
CAMK4	0,0283	0,068
JCAD	0,0283	0,068
WDR82	0,0283	0,068
WNT4	0,0283	0,068
COLEC12	0,029	0,068
RNF217	0,029	0,068
TFAP2B	0,029	0,068
NPTN	0,0298	0,068

STARD8	0,0298	0,068
COPS8	0,0306	0,068
TNFRSF10D	0,0313	0,068
BTD	0,0321	0,068
NCMAP	0,0321	0,068
NIN	0,0321	0,068
RPL27A	0,0321	0,068
SLITRK4	0,0321	0,068
THOC5	0,0321	0,068
VEZT	0,0328	0,068
DYRK1A	0,0336	0,068
HLA-B	0,0336	0,068
NOTCH1	0,0336	0,068
GOLIM4	0,0343	0,069
IER3IP1	0,0343	0,069
SLC1A2	0,0358	0,0705
TACR3	0,0366	0,0711
C7orf55-LUC7L2	0,0374	0,0713
NAP1L1	0,0389	0,0718
DGKH	0,0396	0,0718

EIF1	0,0396	0,0718
LUC7L2	0,0404	0,0718
SUPT16H	0,0404	0,0718
SYT2	0,0419	0,0718
YWHAH	0,0419	0,0718
HSPA6	0,0434	0,0727
SLC25A37	0,0434	0,0727
FAM104A	0,0449	0,0739
CLSTN1	0,0472	0,0755
FOXP1	0,0472	0,0755
STMN1	0,0472	0,0755
EOGT	0,0487	0,0757
GALNT10	0,0487	0,0757
LSM14A	0,0494	0,0757
